# Comparison of the Effect of High and Low Doses of Adrenocorticotropic Hormone (ACTH) in the Management of Infantile Spasms

**Published:** 2020

**Authors:** Afshin FAYYAZI, Reihane ESLAMIAN, Ali KHAJEH, Maryam DEHGHANI

**Affiliations:** 1 Department of Pediatrics Neurology, Hamadan University of Medical Sciences, Hamadan, Iran.; 2 Department of Pediatrics Neurology, Children and Adolescents Health Research Center, Zahedan University of Medical Sciences, Zahedan, Iran.; 3 Instructor of Pediatrics Nursing, Nahavand School of Allied Medical Sciences, Hamadan University of Medical Sciences, Hamadan, Iran.

**Keywords:** Infantile spasms, ACTH therapy, High-dose treatment, Low-dose treatment, Side effects

## Abstract

**Objectives:**

Infantile spasms can have irrecoverable adverse effects on a child’s brain. Adrenocorticotropic hormone (ACTH) is the most common first-line medication for the treatment of infantile spasms. However, the suitable dose and duration of treatment continue to be debated among specialists. Since high doses of ACTH, which are commonly used, can produce more side effects, lower doses are preferred. The aim of this study was to determine the effect and extent of complications caused by high and low doses of ACTH in children with infantile spasms.

**Materials & Methods:**

This clinical trial was performed on 32 infants with infantile spasms, aged 1.5-18 months. The subjects were divided into high- and low-dose ACTH groups. Treatment continued for two months. The therapeutic effects and complications were then compared over 18 months.

**Results:**

The results indicated no significant difference between the groups in terms of the short-term prognosis of convulsions, final prognosis of patients with spasm relapse, EEG changes after treatment, and post-treatment development of hypertension. On the other hand, there was a significant difference in the frequency distribution of restlessness intensity and becoming Cushingoid, which were more frequent in the high-dose group.

**Conclusion:**

The results indicated that high- and low-dose ACTH are equally effective in controlling spasms, yet the low dose causes fewer side effects.

## Introduction

Infantile spasms are one of the most common convulsive syndromes in childhood (1-3). Children who are diagnosed with these spasms are within the age range of two months to less than one year (4, 5). The peak incidence of these spasms occurs between three and seven months (50-77%), and occurrence is rare after 18 months of age (6). In half of patients, cognitive development is normal until the onset of convulsions, while cognitive delay has been observed in others (7, 8). The approximate incidence of this condition is 2-5 infants out of every 10,000 successful births (9). Infantile spasms at older age evolve into Lennox-Gastaut syndrome; therefore, this epileptic pattern is not observed at older age (10). Etiologically, these convulsions are categorized into symptomatic, crypto-genetic, and idiopathic (11, 12). 

In terms of diagnostic measures, a complete awake and asleep electroencephalography (EEG) can detect the presence of hypsarrhythmia in 50-75% of cases as very high and low voltage waves across all cortical regions (13-15). Magnetic resonance imaging (MRI) is also helpful in diagnosing intracerebral lesions, which can predispose an infant to infantile spasms (16). Mental retardation occurs in 75% of afflicted infants and cerebral palsy in 50% (17). Statistically, five out of every 100 children with infantile spasms do not survive for more than five years (18). Less than half of patients with infantile spasms do not experience any attacks using medications (12). Diagnosis of infantile spasms, especially the cryptogenetic and idiopathic types, is a medical emergency, as delay in diagnosis for three weeks or longer affects the long-term prognosis (19). Therapeutically, various agents are used, but no medication has proven to be 100% effective so far. 

 Corticosteroids are the most common form of treatment for infantile spasm, with adrenocorticotropic hormone (ACTH) being the first-line medication (20, 21). Vigabatrin is the first-line medication for children with tuberous sclerosis and infantile spasms (22). There are controversies regarding the suitable dose and duration of treatment using ACTH among specialists. Since high doses of this drug, which are commonly used, cause more side effects, lower doses are preferred (23, 24). The aim of this study was to determine the effects and side effects of high- and low-dose ACTH treatments in children with infantile spasms.

## Materials & Methods

The subjects were selected among 1.5- to 18-month-old infants with infantile spasms, who were referred to the pediatric neurological clinic of Besat Hospital in Hamadan, Iran since October 20, 2013. A total of 32 infants were included in the study and categorized into two equal groups. The inclusion criteria were a clinical diagnosis of infantile spasms, no use of antiepileptic drugs to treat the infantile syndrome, and family consent to participate in the program. The exclusion criteria were an altered preliminary diagnosis and lack of patient cooperation. 

 The therapeutic protocol was as follows. Patients in the high-dose group received intramuscular (IM) injections of 0.25 mg of ACTH. The low-dose group received IM injections of 0.1 mg of ACTH. ACTH was administered as follows: week 1: once a day; week 2: once every other day; week 3: twice a week; week 4: once a week; and week 5-8: once every two weeks. 

 The patients were assigned to one of the groups through single-blind block randomization sampling. None of the patients had received ACTH treatment before this study. Patients who had been recently diagnosed with infantile spasms were randomly included in one of the groups and received ACTH treatment without delay. All patients were hospitalized for seven days for medical and neural examinations and monitoring the vital signs and blood pressure. Treatment was initiated according to the study protocol with either a high or low dose of ACTH after advising the family and collecting the signed consent forms. Initial testing for electrolytes was also performed for all patients. Metabolic and other specialized tests were performed if required. In addition, EEGs and CT scans or MRIs were conducted for all patients. Measurement of blood pressure and neurological examinations were performed during hospitalization on a daily basis. After discharge from the hospital, the patients were examined once every two weeks for drug side effects. 

 In the first, second, and fourth weeks and after treatment termination, EEGs were carried out, and any changes were recorded as normal (mild), moderate, or severe (hypsarrhythmia). At the beginning and end of treatment, development was examined in the patients and recorded as normal development, mild developmental delay, or severe developmental delay. To collect data, a checklist was prepared at baseline on the patients’ characteristics and treatment-associated variables at each stage. Information was recorded carefully. 

 Response to treatment was classified as complete treatment (complete absence of convulsions) or relative response to treatment (reduction of spastic convulsions by 50% or more compared to the onset of treatment). Three variables were examined as factors affecting prognosis, including developmental changes, relapse of spasms or convulsions, and death of patients. In terms of EEG, improvement was considered to be the absence of hypsarrhythmia. Data were analyzed in SPSS version 20 using descriptive (frequency, percentage, mean, and standard deviation) and inferential (independent sample t-test and Fisher’s exact test) statistics. The significance level was considered to be 0.05.

## Results

In this study, 32 patients were investigated during 18 months. No patient was excluded during the study. Both groups included the same number of patients. In terms of gender, it was found that infantile spasms were slightly more frequent in boys (56%). The mean and standard deviation of infants’ age in the high-dose and low-dose groups were 8.2±2.6 and 5.2±6.6 months, respectively. The results of t-test indicated a significant difference in the mean age of the groups, but no significant difference was found in the onset of infantile spasms or onset of treatment between the two groups. The results of t-test indicated no significant difference regarding the type of infantile spasm (cryptogenic or symptomatic) between the groups.

 The results of this study showed that patients with infantile spasms had a mean age of four months. On average, they were diagnosed and treated after about two months of delay from the onset of symptoms. In terms of clinical type, 59% of the patients were categorized as flexor, 28% as extensor-flexor, and 13% as extensor. Etiologically, 25 (78%) patients had the symptomatic disease, and its prevalence was not significantly different between the groups. The brain imaging results (MRI or CT) showed that 53% of the patients had positive findings, with the most common imaging findings being cerebral atrophy, periventricular leukomalacia, cerebral dysgenesis, and calcification of the basal ganglia. 

 During six months of treatment, the patients were not significantly different regarding the time and duration of hospitalization. Cause of hospitalization was the incidence of severe side effects, such as hypertension, severe restlessness, infection, or relapse of convulsions with inability to control them. No significant difference was observed in the clinical response to treatment and control of convulsions (P=0.16). In 81% of patients, spasms were controlled during the first week of treatment; the difference was not significant between the groups (P=0.16). 

 Three out of 32 patients died during 18 months of the study (mortality rate=9.4%), all of whom belonged to the high-dose ACTH group. Two patients experienced asphyxia (hypoxic ischemic encephalopathy) and developed severe aspiration pneumonia and respiratory distress. One of the patients was a child with Down’s syndrome and infantile spasms, who died due to sepsis. Although these three patients were in the high-dose group, the frequency of death was not significantly different between the groups (P=0.1). 

 There was no significant difference in development after treatment (P=0.28). By the end of the therapeutic protocol, spasms and convulsions were completely controlled in 18.7% of patients without any need for further medications. In 5.37% of patients, in addition to ACTH, another drug was also used to control the convulsions completely. In 25% of patients, spasms and convulsions were relatively controlled with the addition of another drug. In 3.9% of patients, despite concurrent treatment with ACTH and several other medications, spasms and convulsions were resistant to treatment. No significant difference was observed in neither of four levels defined for controlling convulsions and spasms. 

 There was no significant difference in the time required to control the convulsions. The results indicated that there was a significant difference in EEG recordings before and after treatment. The findings showed that 43% of patients in the low-dose group and 56% of patients in the high-dose group experienced diminished EEG intensity after treatment. Irrespective of drug dose, treatment with ACTH was clearly effective in improving the EEG results (P=0.008) (Table 1). There was no significant difference in EEG changes after treatment between the groups. There was also no significant difference in the incidence of hypertension throughout the treatment (P=0.5). On the other hand, there was a significant difference in the incidence of restlessness and the associated sleep disorders (P=0.002). There was also a significant difference in terms of the incidence of becoming Cushingoid (P=0.04).

## Discussion

Previous studies have shown that use of high-dose ACTH is associated with more side effects and less desirable therapeutic outcomes, compared to its lower doses (25-27). The present study was conducted to investigate the therapeutic effects of high- and low-dose ACTH on the management of infantile spasms in Iranian children. The results of the present study showed that high- and low-dose ACTH were equally effective in controlling spasms. The spasms were controlled in about 81% of patients during the first week of therapy. During the follow-up, about 80% of controlled cases experienced secondary convulsive relapses, but this was not significantly different between the groups. 

**Table 1:** Frequency distribution of treatment groups with ACTH in terms of electroencephalography (EEG) status before and after treatment

**EEG changes**	**Before treatment**		**After treatment**	
	Frequency	Percentage	Frequency	Percentage
Normal	0	0	9	28.1
Mild changes	12	37.5	13	40.6
Severe changes	20	62.5	10	31.3
Total	32	100	32	100

**Table 2:** Frequency distribution of seizure control and spasm in infants at the onset of treatment

	**Days**	**Weeks**	**Without control**
Low-dose ACTH	15	1	0
High-dose ACTH	11	3	2

**Table 3:** Frequency distribution of treatment groups with high- and low-dose ACTH according to the state of development after treatment

	**Normal**	**Developmental delay**
Low-dose ACTH	3	13
High-dose ACTH	1	15

**Table 4.** Distribution of seizure outcomes and relapse in infants after treatment

	**Control with ACTH**	**Control with other medications**	**Relative control**	**Relapse**	**Death**
Low-dose ACTH	4	7	3	2	0
High-dose ACTH	2	5	5	1	3

In a study by Hrachovy et al. (1994), 13 out of 30 patients treated with high-dose therapy experienced complete cessation of spasms, and of 29 participants treated with low-dose therapy, 14 experienced complete cessation of spasms (25). In a study by Yanagaki et al. (1999), 11 out of 13 patients treated with high-dose ACTH and 9 out of 13 patients treated with low-dose ACTH experienced spasm cessation (26). These two studies revealed that high-dose ACTH ended the spasms in 79.5% of patients versus 76.5% of patients with low-dose ACTH. There was no significant difference in terms of relapse rate between the two groups (14). Moreover, in a study by Zeng et al. (2011), there was a trend towards more complete cessation of spasms in the low-dose ACTH group, although the difference was not statistically significant. Relapse was more frequent in the low-dose ACTH group, but no significant difference was found between the groups (28).

Response to treatment and final prognosis of development were better in patients with cryptogenetic infantile spasms, which is consistent with the results of previous studies (29). In terms of gender distribution, it was observed that infantile spasm is slightly more frequent in boys, which is in line with the results of most previous studies (25, 30). This phenomenon can be attributed to the presence of a genetic predisposition, including the *ARX* gene in boys, which is related to the higher percentage of male patients. 

 The results showed that patients in both groups experienced infantile spasms at the mean age of about four months. They were diagnosed and treated after about two months of delay. This delay has been also observed in other studies and is associated with vague symptoms of the disease for families and general practitioners. EEG changes after treatment in both groups suggest that ACTH improved the EEG quality in infantile spasms, although different drug doses did not affect the extent of changes between the groups; this finding is in accordance with the results of previous studies (26, 27, 31). In this regard, Zeng et al. (2011) found no significant difference in the disappearance of hypsarrhythmia between the high-dose and low-dose groups (28).

 The present results revealed that there was a significant difference between the groups in terms of the incidence of hypertension. There was also a significance difference in the incidence of restlessness and the associated sleep disorders. Based on the findings, 83% of patients, who experienced severe restlessness and visited a physician, belonged to the high-dose ACTH group. There was also a significant difference in becoming Cushingoid between the groups, with more high-dose patients becoming Cushingoid. There was no significant difference between the groups regarding the time and duration of hospitalization six months after the initiation of treatment. About 90% of patients showed further development of the disorder by the end of the follow-up, which was similar in both groups. Three patients died during treatment, all of whom belonged to the high-dose ACTH group with symptomatic spasms. Although these three patients belonged to the high-dose group, the difference in death frequency was not significant between the groups.

**In conclusion**, the results indicated that high- and low-dose ACTH are equally effective in controlling spasms. Lower doses of ACTH did not result in the further relapse of convulsions, but it was found that use of higher doses increased the incidence of side effects. Lack of difference in the therapeutic outcomes between the groups, along with the reduction of side effects in the low-dose group, justifies the use of low-dose ACTH for controlling infantile spasms.
